# Large-scale preparation of Co nanoparticles as an additive in carbon fiber for microwave absorption enhancement in C band

**DOI:** 10.1038/s41598-021-81848-7

**Published:** 2021-01-26

**Authors:** Y. X. Zhu, S. F. Wang, Y. S. Zhang, Z. G. Wu, B. Zhong, D. R. Li, F. Y. Wang, J. J. Feng, J. Tang, R. F. Zhuo, P. X. Yan

**Affiliations:** 1 School of Physical Science and Technology, Lanzhou University, Tianshui Road, Lanzhou, 730000 China; 2 Institute of Nanomaterials Application Technology, Gansu Academy of Science, Dingxi Road, Lanzhou, 730000 China; 3 School of Materials Science and Engineering, Harbin Institute of Technology at Weihai, Weihai, 264209 China

**Keywords:** Materials science, Nanoscale materials, Electronic properties and materials, Magnetic properties and materials, Nanoparticles

## Abstract

Recent studies have found that the core–shell structured metal nanoparticles and porous carbon nanofibers (PCNF) are combined into a microwave absorbing material through electrospinning, which exhibits excellent microwave absorption performance. In this study, the core–shell structure Co nanoparticles prepared by the self-developed HEIBE process (production rate of > 50 g/h) were combined with porous carbon fibers, and their absorbing properties were greatly improved. The morphology of Co/PCNF demonstrated that CoNPs are randomly dispersed in the porous carbon nanofibers and carbon nanofiber form complex conductive network which enhances the dielectric loss of the materials. Meanwhile, the Co/PCNF has a low graphitization and shows a significant improvement in permittivity due to the combination of CoNPs and high conductivity of carbon material. The maximum reflection loss (RL) of Co/PCNF reaches − 63.69 dB at 5.28 GHz with a thickness of 5.21 mm and the absorption bandwidth (RL ≤  − 10.0 dB) is 12.92 GHz. In terms of 5.60 mm and 6.61 mm absorber, there are two absorption peaks of − 47.64 dB and − 48.30 dB appear around 12.50 GHz and 14.10 GHz, respectively. The results presented in this paper may pave a way for promising applications of lightweight and high-efficiency microwave absorbing materials (MAMs).

## Introduction

With the explosive advance of novel electronic, wireless, and radar devices, which has led to the frequent occurrence of such safety problems as human health, national defense security, and electronic safety, etc^1^. Microwave absorbing materials (MAMs) can effectively eliminate electromagnetic waves through electrical loss, magnetic loss, and impedance matching^2,3^. The single absorber has disadvantages that can’t meet the requirements of advanced MAMs, such as narrow absorption band, weak reflection attenuation, thick coating, and high density^4,5^. Therefore, many studies in the past have focused on the combination of magnetic material and carbon material, such as Co-CNTs^6^, Co/C nanoparticles^7^, Co/C nanocomposites^8^,Co/Graphene^9^, and Co/C-800^10^, and the purpose is effectively evaluating and calculating absorption properties by adjustment the relative complex permittivity (ε_r_ = ε′ − jε″) and permeability (μ_r_ = μ′ − jμ″). However, the maximum absorption value of this type of absorber is mainly distributed over a relative narrow frequency range, and this will limit the practical application of lightweight and high-performance MAMs.

Carbon materials have been widely used in microwave absorbing materials because of their large specific area, low cost, good stability, low cost, and good electrical conductivity. Carbon materials can be classified into carbon fiber, carbon nanotubes, carbon black, graphene, and porous carbon. Noticeably, porous carbon fibers compared with traditional carbon fibers, it has many advantages such as lighter quality, possess many holes on the surface of the fiber, and more excellent electrical conductivity^11–13^. The porous structure of porous carbon fibers will improve the stability of MAMs. At the same time, due to the existence of a large number of heterogeneous interfaces between different components, the structure between carbon nanoparticles and magnetic nanoparticles can enhance the polarization of the interface^14^. Among typical magnetic metals (Fe, Co, and Ni), cobalt nanoparticles can be considered as ideal magnetic filler candidate, because it has stable chemical properties, large saturation magnetization, strong anisotropy field and high Snoek's limit in the GHz range^15^.

In this paper, core/shell structure cobalt nanoparticles (CoNPs) were preparation of macro and added to polyacrylonitrile/N,N-Dimethylformamide (PAN/DMF) solution containing vegetable oil to prepare Co/C porous nanofibers (Co/CPNFs) by electrospinning. To the best of our knowledge, there are a few reports about the combination of porous carbon fibers and magnetic metal nanoparticles. Therefore, in view of these advantages of porous carbon fiber, this work focuses on the synthesis and microwave absorption properties of Co/PCNF. The Co/PCNF have excellent microwave absorption properties with the maximum absorption value of − 63.69 dB at 5.28 GHz. Such good absorbing properties are attributed to the combined action of impedance matching, dielectric loss, magnetic loss, and attenuation constant. This work provides a novel strategy to achieve the production of MAMs, which has lightweight, strong absorption, wide absorbing band.

## Experimental section

### Preparation and synthesis

#### Preparation of the magnetic cobalt nanoparticles

The CoNPs were produced by a method called HEIBE, which was mentioned in our previous report^16^. In simple terms, the preparation process of CoNPs is as follows: Use ion beam to bombard the cobalt block in a sealed environment with a vacuum of 10^−3^ Pa. When the heat energy on the surface of the cobalt block reaches a certain level, a large amount of cobalt vapor will be generated in the vacuum chamber. The condensing cycle system is then used to quickly cool the cobalt vapor so that it nucleates and crystal grains grow. The oxygen (5 sccm) was passed into the closed chamber to passivate the obtained CoNPs, and an oxide layer was formed on the surface of the CoNPs. Ultimately, the core–shell structure of CoNPs were collected owing to the rapid homogeneous nucleation and quenching.

#### Synthesis of the Co/porous carbon nanofibers

**Figure 1:**
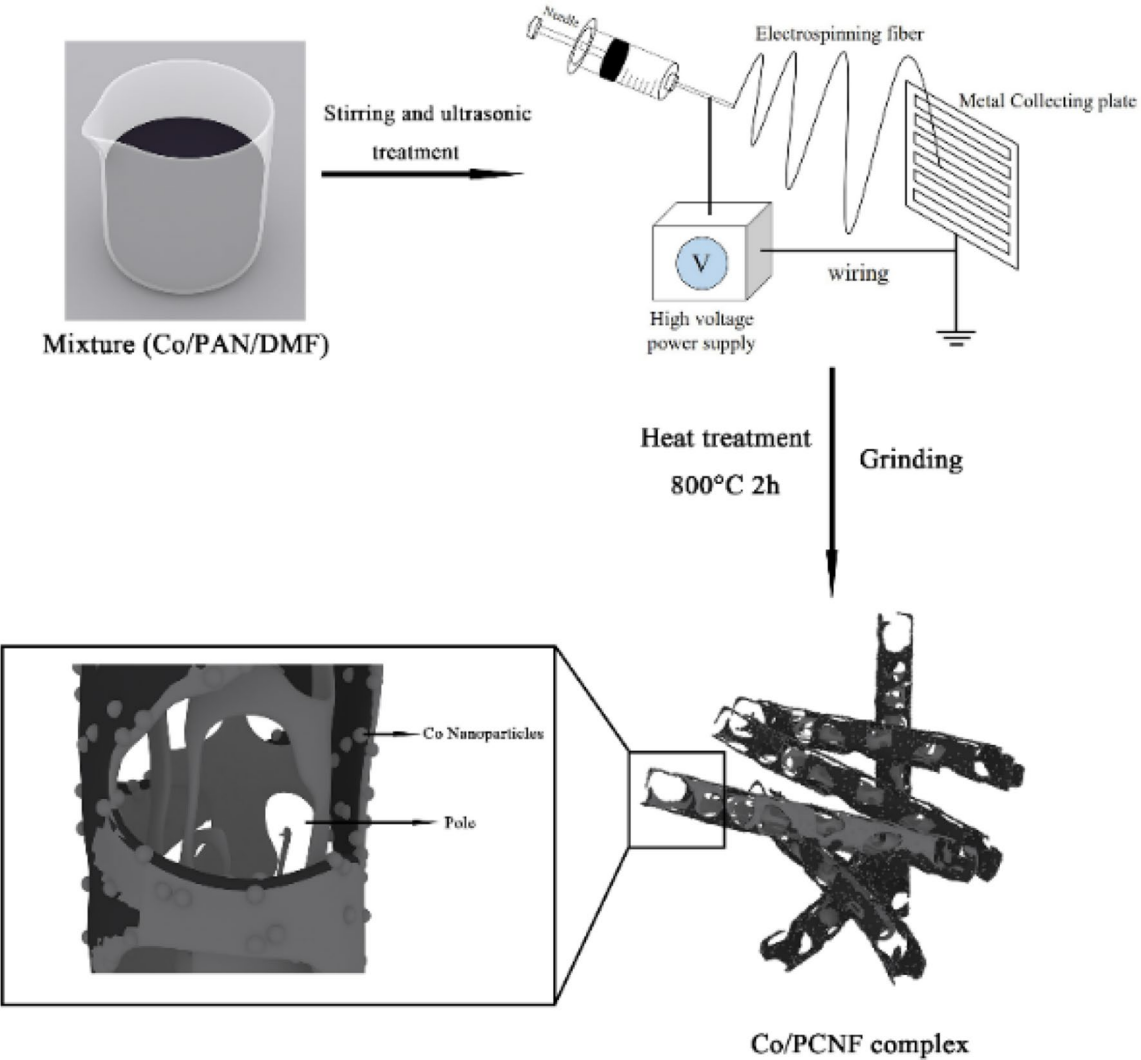
The schematic illustration of the fabrication process of Co/PCNF.

the Co/PCNF were synthesized by electrospinning based on an earlier paper^16,17^, which is as follows: Firstly, 0.8 g of PAN (Mw = 150,000) was dissolved in 10.0 g of DMF by magnetic stirring at room temperature, then 1.08 g of vegetable oil and CoNPs were added to the above mixture, and mechanically stirred at room temperature for 12 h to obtain a stable suspension. The obtained spinning solution was subjected to ultrasonic treatment for 15 min, and then the spinning solution was transferred to the disposable syringe for electrostatic spinning. The conditions of electrospinning are as follows: the work distance was 22 cm, and the work voltage was 30 kV. The feed rate was 30 µL/min. The product is collected by a collector composed of grounded stainless steel strips (the width between the steel strips is 1 cm), and the spinning process Always keep the surface temperature of the collector around 80 °C. The gray product on the collector was placed in a tube furnace at a heating rate of 2 °C/min at 800 °C for 2 h, and argon gas (50 sccm) was introduced during the heat treatment. Name the obtained sample S3, and repeat the experiment with the added amount of CoNPs (0.05 g, 0.26 g), and name the final samples S1 and S2 respectively. Figure 1 illustrated a simple schematic diagram of the synthesis of magnetic Co/porous carbon nanofiber and how electromagnetic wave attenuation comes about.

### Characterizations

The crystal phase is recorded on X-ray powder diffraction (XRD, X'pert powder, Philips), while using Cu K_α1_ (λ = 1.540 Å) radiation. The microstructure and morphology are determined using scanning electron microscopy (FIB-SEM, Tescan LYRA 3 XMU), and scanning transmission electron microscopy (STEM). The shape and size of the particles were determined from high-magnification transmission electron microscopy (HRTEM, FEI Tecnai G^2^ F30). The degree of graphitization was confirmed by Micro-Raman spectroscopy (Jobin–Yvon Horiba HR800) using argon ion laser (k = 532 nm) in the range of 800–1800 cm^−1^. The pore-size distribution is investigated by Brunauer–Emmett–Teller measurement (BET, ASAP 2020 Micromeritics), and the magnetic properties were carried out by vibrating sample magnetometer (VSM Lake-Shore 7404, USA) in room temperature.

The EM parameters are analyzed in the test range (2.00–18.00 GHz) by using a vector network analyzer (VNA, Agilent N5245A). According to the mass ratio of sample to paraffin of 1:5, the sample are pressed into a toroidal shape (outer diameter of 7.0 mm and inner diameter of 3.04 mm). The calculated reflection loss (RL) and simulated reflection loss (RL) are calculated by the coaxial reflection/transmission method based on the NRW method and the transmission line theory, respectively.

## Results and discussion

### Structure and morphology analysis

**Figure 2:**
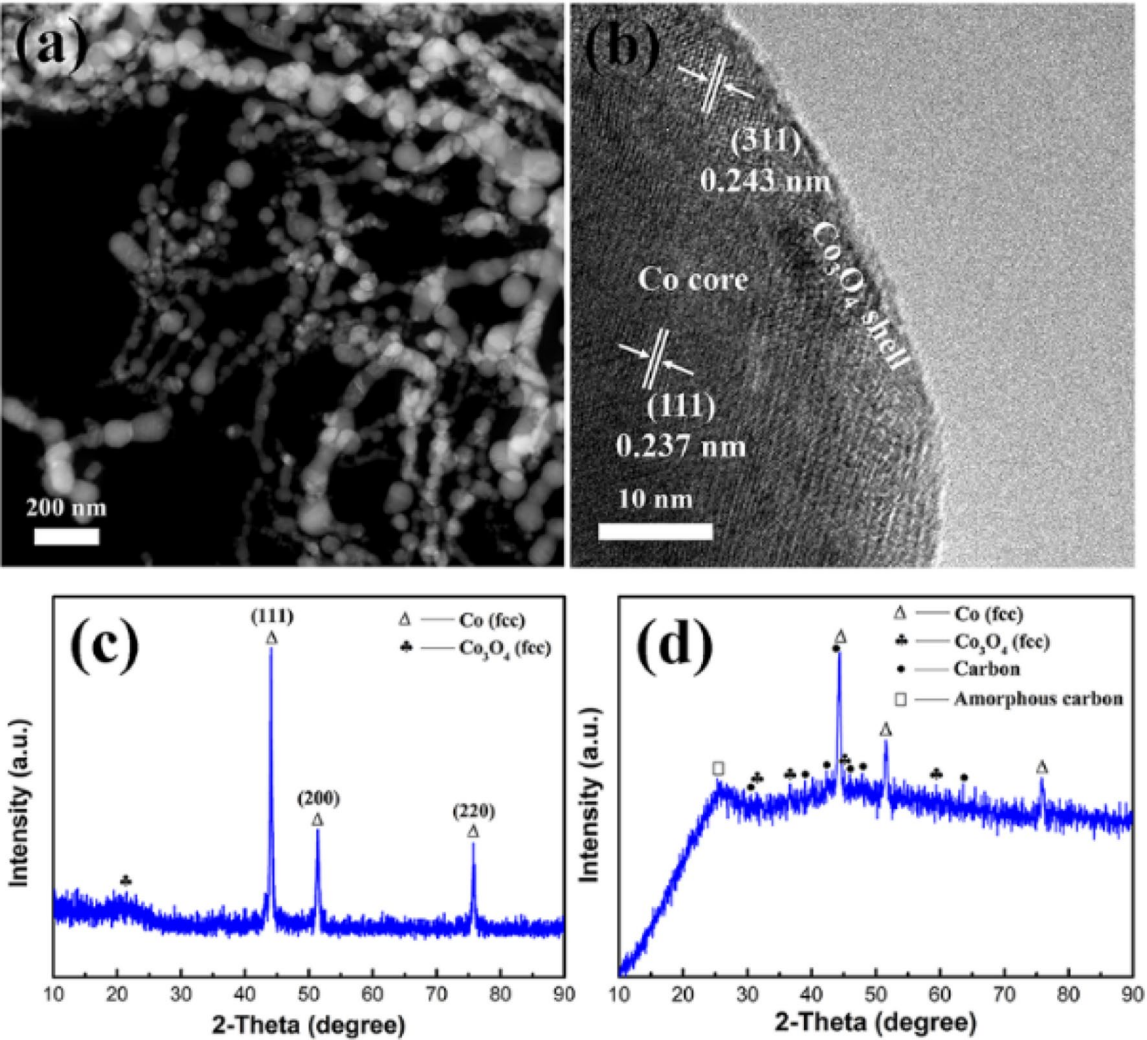
(**a**) STEM image, (**b**) HRTEM image, (**c**) XRD pattern of CoNPs, and (**d**) XRD pattern of Co/PCNF.

The CoNPs have been produced massively at a production rate of > 50 g/h, the carbon nanofibers were synthesized by electrospinning followed stabilization and carbonization. The phase composite measured by XRD of Co/PCNF is shown in Fig. 2c, XRD pattern of CoNPs indicates the CoNPs consist of fcc structure Co (JCPDS 15-0806) and Co_3_O_4_ (JCPDS 43-1003). Based on JCPDS card No. 15-0806, three diffraction peaks of cobalt centered at 2θ = 44.2°, 52.4°, and 76.4° are corresponded to (111), (200), and (220) planes, respectively. The sharp diffraction peaks and high intensity mean that the magnetic Co core has highly crystallized. Besides, we can observe a very weak peak of Co_3_O_4_ coating. This result is in accord with the HRTEM test results represented in Fig. 2b. Using the Scherrer equation: D = kλ/β_1/2_cosθ^18^, where D is the average crystallite size, k a shape function for which a value of 0.9 is used, λ the X-ray wavelength in nanometer (nm), β_1/2_ the full-width at half maximum (FWHM) of the strongest reflection peak (111); thus, the average grain size of CoNPs is approximately 100 nm. From STEM image (Fig. 2a), it can be seen that CoNPs are spherical in shape and some particles connected in series. Simultaneously, this result is also in good agreement with the XRD results. The HRTEM image (Fig. 2b) exhibits that CoNPs are core/shell structure, with a thin shell (2–5 nm), and combined with results of XRD, we can know that the out layer of particles is cobalt oxide and core is cobalt element. Furthermore, the lattice fringe of the outer shell is corresponding to (311) crystal plane of Co_3_O_4_, the lattice fringe of the core is corresponding to (111) crystal plane of Co. The XRD pattern of Co/PCNF is shown in Fig. 2d. Compared to CoNPs, in addition to the peaks of Co and Co_3_O_4_ in Co/PCNF, there is also a visible peak in 25.2°, indicating the annealed Co/PCNF contains amorphous carbon, it will be demonstrated again in the Raman test results.

**Figure 3:**
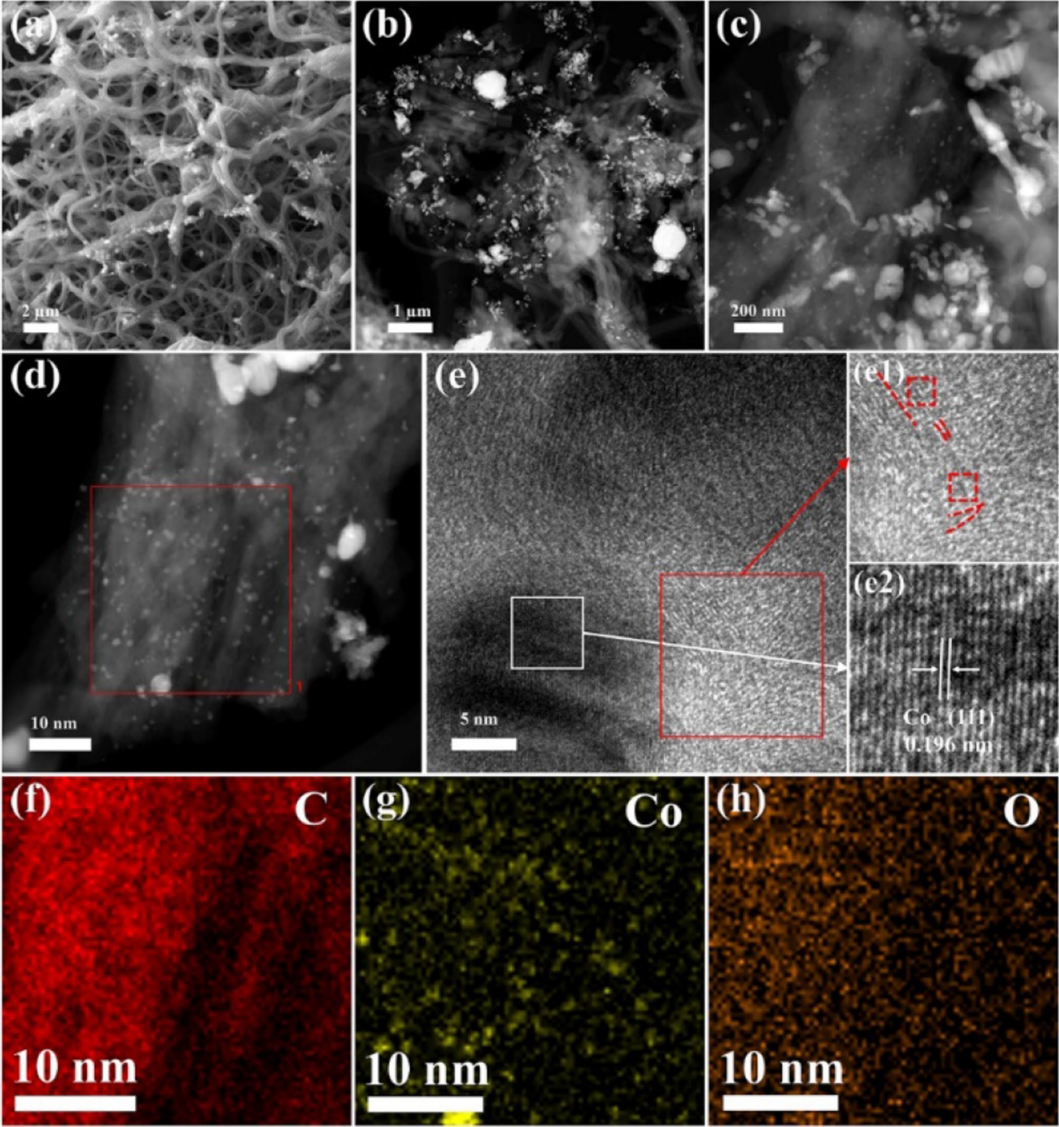
SEM images (**a**–**c**) under different magnification, (**d**) STEM image, (**e**) HRTEM image of Co/PCNF; (**e1**) crystal plane of the carbon around the pore and (**e2**) crystal plane of CoNPs particles embedded in the samples; elemental mapping of Co/PCNF in some area of Co/PCNF: (**f**) carbon mapping; (**g**) cobalt mapping; (**h**) oxygen mapping.

The morphology and microstructure of Co/PCNF can be investigated by SEM and TEM. The SEM image (Fig. 3a) indicates that Co/PCNF have 3D cross-linked network structure with entanglement, the average diameter of the fiber ranges from 100 to 500 nm and many pores are distributed on the surface of the fibers. In addition, there are many entanglements among nanofibers because of irregular entanglement behavior of PAN after oxidation stability. From STEM images as shown in Fig. 3b,c, it can be seen that the particles with a size less than 30 nm are embedded in the fibers. Instead, the larger particles are agglomerated around the fiber surfaces. As shown in Fig. 3d, the Co nanoparticles are very uniformly dispersed in the carbon fiber, which is due to the long-term ultrasonic treatment and stirring during the preparation of the material. Figure 3f,h demonstrate the elemental mappings of Co/PCNF, the results show that the compound contains elements Co, C, and O. Among them, the Co, O are evenly distributed inside the distribution area of carbon. These consequences furtherly confirm that the pores with different sizes are distributed randomly in the carbon nanofiber. Besides, the monodisperse CoNPs are distributed on the surface or inside of the porous carbon matrix homogeneously, it is due to long time stirring and ultrasonic treatment. To explore the existence of cobalt nanoparticle in carbon fibers and the phase structure of carbon, the HRTEM images (Fig. 3e, e1) show a part of the crystallization of carbon materials, but the lattice distortion is not perfect, such as atom deletions, dislocations and lattice fringes bending. Figure 3e2 shows a typical Co lattice fringe, the result corresponding to the XRD. Above all, we think that the existence of particles and pores is the main cause of the defects in this composite material, which is be beneficial to the improvement of absorbing performance. After further estimating the mass percentage and atomic percentage of each element in S2 by an energy spectrometer, the results are shown in Table 1. It can be seen that C is the main component element in the sample, and Co content is low. Therefore, it can be concluded that in the case of a small amount of CoNPs prepared by the HBIE method, the absorption performance of the absorbent can be significantly improved while the density of the absorbent is kept low.

**Table 1:** X-ray energy spectrum analysis result of Co/PCNF (S3).

Element	Mass percentage	Atom percentage
C	93.96	97.5
O	2.16	1.68
Co	3.88	0.82
Gross	100	100

**Figure 4:**
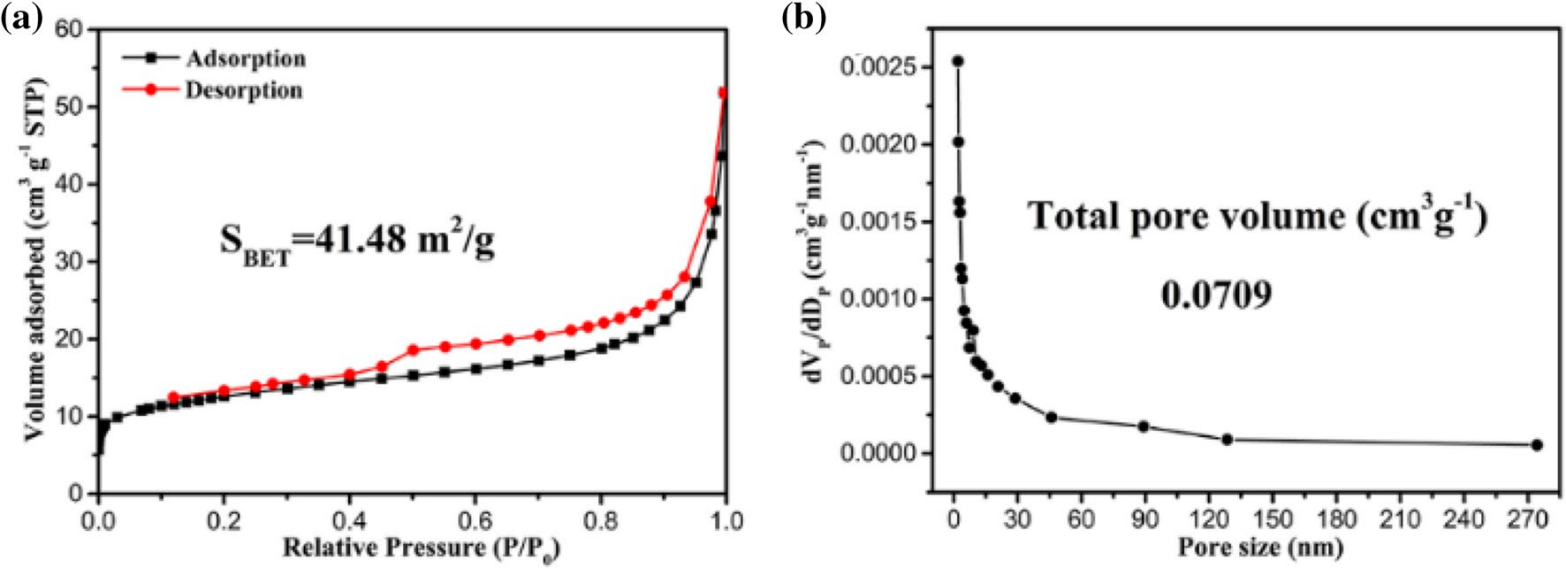
(**a**) Nitrogen adsorption–desorption isotherms and (**b**) pore size distributions of Co/PCNF.

Nitrogen adsorption/desorption was used to characterize the porous carbon fiber in order to further comprehend the microstructure of pore. The specific surface area and pore size distribution of Co/PCNF were represented by nitrogen BET as shown in Fig. 4. The S_BET_ of Co/PCNF is 41.48 m^2^/g and the total pore volume is 0.0709 cm^3^/g^−1^. The formation of porosity is based on the sacrifice template method^19^.

### Raman study and magnetic properties

**Figure 5:**
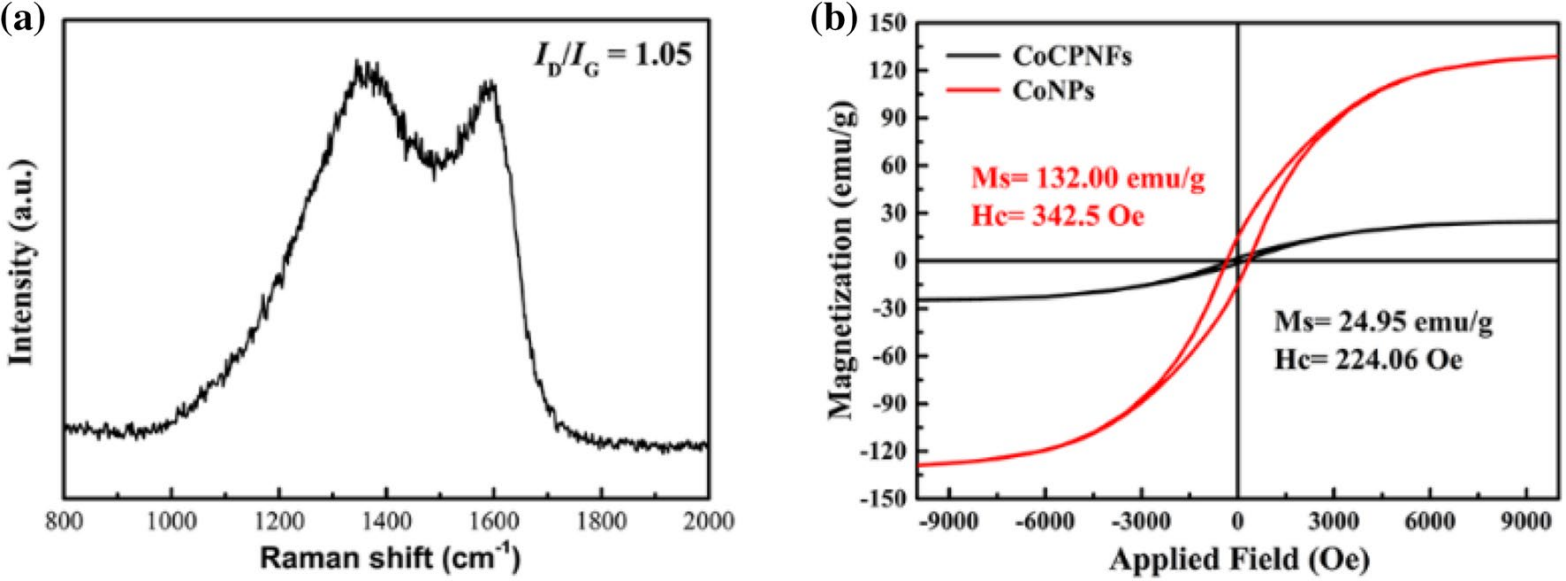
Raman spectrum (**a**) of Co/PCNF; hysteresis loop (**b**) of CoNPs and Co/PCNF at room temperature.

The banding state of carbon (graphitization degree) in Co/PCNF can be further studied by Raman spectra^20^. Figure 5(a) displays the Raman spectra of Co/PCNF. As reported^21^, the two broad peaks in the graph represent amorphous carbon (D-band), it is ascribed to the vibrations of sp^3^ carbon atoms of disordered graphite, indicating the amorphization degree of samples, and graphitization carbon (G-band), it is related to the in-plane vibration of sp^2^ carbon atoms in a 2D hexagonal lattice, which is related to graphite carbon, respectively. The most obvious characteristics of Raman spectroscopies are the D and G bands at 1350 cm^−1^ and 1580 cm^−1^, separately. It can be seen that the intensity ratio (*I*_D_/*I*_G_) of D band (1363 cm^−1^) and G band (1594 cm^−1^) is 1.05. The degree of graphitization is evaluated by *I*_D_/*I*_G_^7^, this result indicates Co/PCNF contain a certain amount of structural defects and have a low graphitization degree. We speculate that the reason for the low degree of graphitization of Co/PCNF is that the presence of CoNPs and pores makes carbon form visible small crystals around it (as shown in Fig. 3e2). Because they are disordered and not perfect with crystal imperfection, such as, vacancy, interplanar spacing widening and crystal bending, etc., resulting in a low degree of Co/CNF graphitization. In addition, CoNPs has good dispersion and is surrounded by crystal carbon, which improves the corrosion resistance of CoNPs and establishes a good electromagnetic matching. Based on previous research results, the relative low graphitization degree will be favorable for improving the impedance matching of microwave absorbing materials^7^.

To reveal the microwave absorption mechanism of composite materials, Fig. 5b shows the magnetic properties at room temperature for CoNPs and Co/PCNF. The hysteresis loop shows that the samples have a strong magnetic response under varying external magnetic field. The saturation magnetization (M_s_), the coercivity (H_c_) values of CoNPs and Co/PCNF are 132.00 emu/g, 24.95 emu/g, and 224.06 Oe, 342.50 Oe, respectively. Ferromagnetic MAMs possessing high initial permeability (μ_i_) which decides it has a stronger magnetic loss, and initial permeability (μ_i_) can be defined by the equation as follows^15^:

1 $$\upmu _{{\text{i}}} { = }\frac{{{\text{M}}_{{\text{S}}}^{{2}} }}{{{\text{akH}}_{{\text{c}}} {\text{M}}_{{\text{s}}} {\text{ + b}}\uplambda \upxi }} $$

where a and b are two constants associated with absorber, λ the magnetization constant, ξ the elastic strain parameter of the crystal, M_s_ the saturation magnetization and H_c_ the coercivity. It is reasonable that larger M_s_ and smaller H_c_ are the two basic conditions of higher initial permeability (μ_i_)^22^. It can be seen from Fig. 5b that CoNPs have larger M_s_ and smaller H_c_ in the case of external magnetic field, and this will enable CoNPs own a larger initial permeability (μ_i_). Therefore, CoNPs has better magnetic loss ability compared to Co/PCNF, but the too high magnetic loss will lead to unbalance matching impendence and decrease of microwave absorbing performance, which will be explained in detail later.

### Elemental analysis

**Figure 6:**
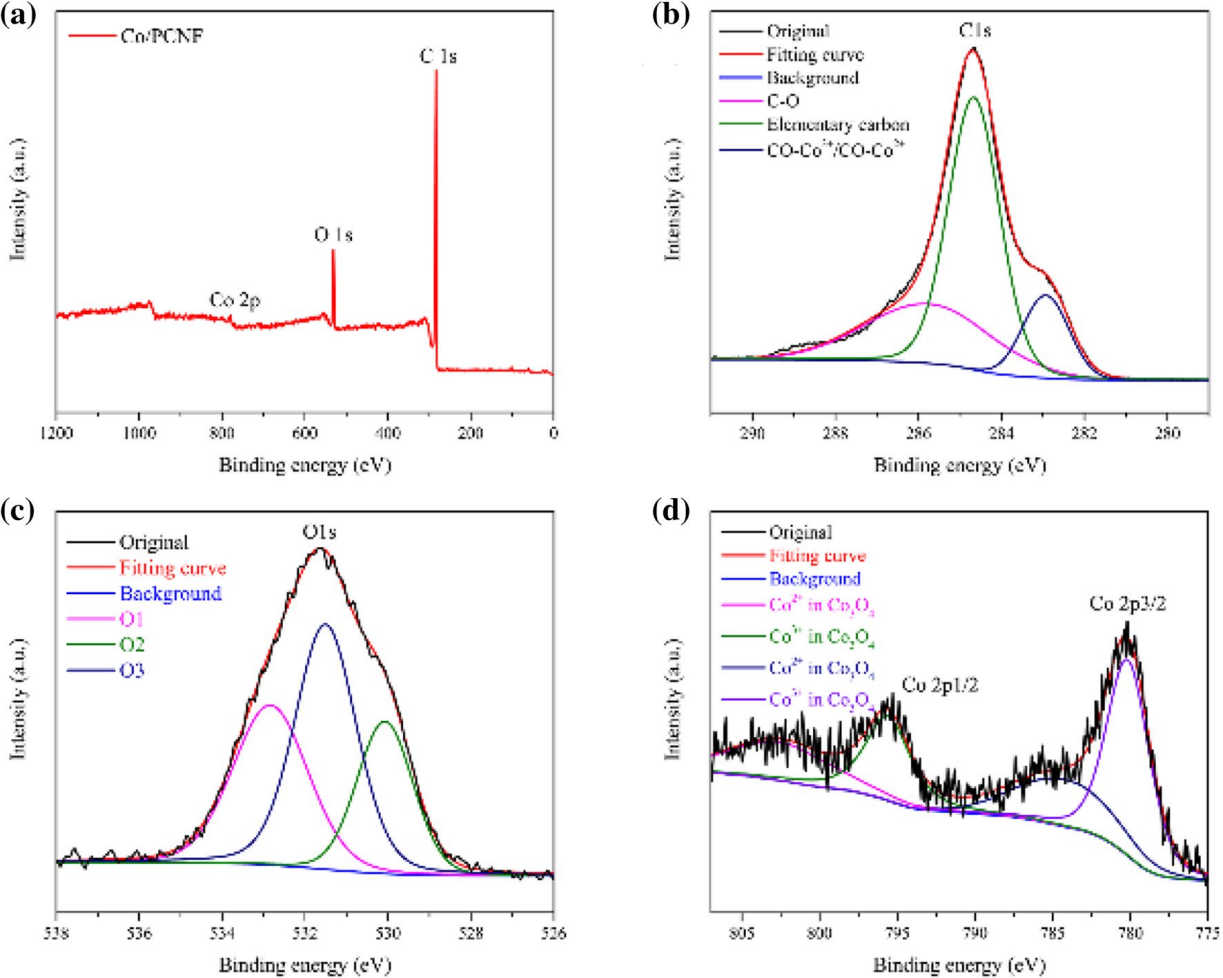
Raw scan XPS spectra (**a**) of Co/PCNF. XPS survey spectrum and deconvolution of Co/PCNF (S1) (**b**) C 1s, (**c**) O 1s, (**d**) Co 2p.

X-ray photoelectron spectra (XPS) and X-ray absorption fine structure (XAFS) further analyzed the surface composition of Co/PCNF and the valence states of C, Co, O, as shown in Fig. 6. Figure 6a shows the XPS full spectrum of Co/PCNF, where C, Co and O signals are clearly observed, which means that the sample is mainly composed of C, Co, O and there are no other impurity elements. This result is consistent with the experimental data of XRD. The C 1s spectrum (Fig. 6b) can be regarded as the fitting corresponding to the three photoemission peaks of CO–Co^2+^/CO–Co^3+^, elementary carbon and C–O, located at 282.9 eV, 284.6 eV and 285.7 eV, respectively^56^. As shown in Fig. 6c, the O 1s of Co/PCNF show three characteristic peaks located at 532.8 eV (noted as O1), 530.1 eV (noted as O2) and 531.5 eV (noted as O3), which can be assigned to hydroxyl groups or surface-adsorbed, oxygen lattice oxygen in pristine Co_3_O_4_ and vacancy sites with a low oxygen coordination, respectively^57^. For the XAFS spectrum of Co, the two fitting peaks of Co2p^3/2^ and Co2p^1/2^ are composed of Co^3+^ and Co^2+^, and the area that the fitted curve covered shows the relative atomic ratio of surface Co^2+^/Co^3+^. The ratios of Co^2+^/Co^3+^ of the Co/PCNF is 0.65, which is greater than the value of Co^2+^/Co^3+^ of pure Co_3_O_4_. This may be the reason for the deviation of the Co 2p peak of Co/PCNF from the Co 2p peak of pure Co_3_O_4_^56,57^. According to the above results, it can be concluded that Co_3_O_4_ exists in Co/PCNF, which is consistent with the XRD results. In addition, through electrospinning and heat treatment, a chemical bond is formed between CoNPs and the shell (Co_3_O_4_) of PCNF.

### Microwave absorption property

**Figure 7:**
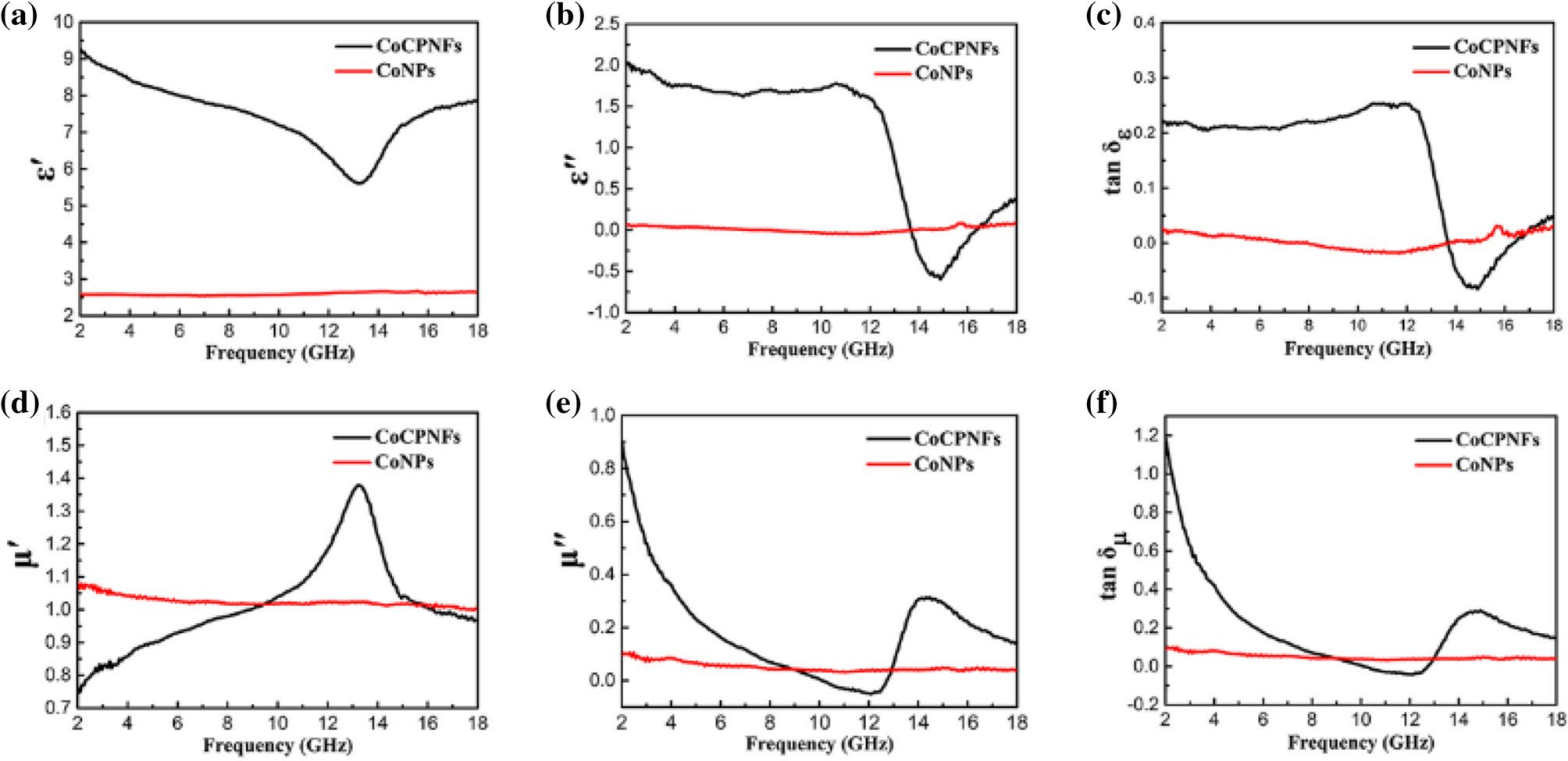
Frequency dependence of (**a**) real parts (ε′) and (**b**) imaginary parts (ε″) of complex permittivity; (**d**) real parts (µ′) and (**e**) imaginary parts (µ″) of complex permeability; (**c**) dielectric loss (tanδ_ε_) and (**f**) magnetic loss (tanδ_µ_) for CoNPs and Co/PCNF.

In order to further explore the microwave absorption characteristics of Co/PCNF, two important parameters, the complex permittivity (ε_r_ = ε′ − jε″) and complex permeability (μ_r_ = μ′ − jμ″), were determined by a vector network analyzer^23^. Specifically, the real part of permittivity ε′ (and permeability μ′) are related to the storage of electromagnetic energy and the imaginary part of permittivity ε″ (and permeability μ″) represent the loss of electromagnetic energy^24,25^. Two basic electromagnetic parameters (ε_r_ and μ_r_) of CoNPs and Co/PCNF in 2.0 − 18.0 GHz are shown in Fig. 7a,b,d,e. As illustrated in Fig. 7a,b, the complex permittivity of Co/PCNFs, as the frequency increases, both the real part (ε′) and the imaginary part (ε″) decrease slightly in 2.0–10.0 GHz. It’s worth noting that this phenomenon of ε′ and ε″ drops sharply in the high-frequency range (10.0–14.5 GHz) is called frequency dispersion behavior, which is found in most carbon materials, such as carbon nanotubes (CNT), carbon black, graphene, and porous carbon. Moreover, the appearance of strong resonance peaks of ε′ and ε″ may be associated with the interfaces between Co cores and lattice defects, because it may lead to the interfacial polarization and interfacial relaxation (as show in Fig. 10b)^26^. Compared with Co/PCNF, the values of ε′ and ε″ of CoNPs are fluctuating up and down in the vicinity of 2.6 and 0.05 respectively in whole range of test frequencies, this phenomenon should be caused by the combination of the inherent dipole orientation polarization in the sample, the interface polarization between the Co particle core and the graphite carbon shell, and the space charge polarization between adjacent Co particles. As can be observed in Fig. 7a, the ε′ value of Co/PCNF is much larger than that of CoNPs, suggesting the CoNPs have poor polarization performance. In addition, the addition of porous carbon nanotubes increases the conductivity of the composite material and provides an additional conductive path for electron hopping and migrating, as shown in Fig. 10a, increase the conductivity loss. The presence of more free electrons in the material means that it can transform high electromagnetic energy, which will be resulted in the increase of the ε′ value^21^. Not only that, compared with the carbon fiber composites without porous structure, the slightly inferior of permittivity for carbon fiber with porous may be due to introduce into the low dielectric air by porous. According to free electron theory in which ρ = 1/πf ε″ε_0_^27,28^, where ε_0_ is a constant of 8.854 × 10^–12^ F/M, ρ is the resistivity of samples. It can be known that the higher value of ε″ means lower ρ value. From 2.0 to 13.7 GHz (shown in Fig. 7b), the ε″ value of Co/PCNF is higher than that of CoNPs, indicating CoNPs has a very poor dielectric loss. For Co/PCNF, we should notice that the negative value exists of ε″ in the frequency range of 13.7–16.5 GHz, meaning the Co/PCNF have no dielectric loss under this circumstance^29^.

The μ′, μ″ of CoNPs show negligible change from 2 to 18 GHz, and especially the μ″ is close to zero in entire frequency range. In contrast, when the Co nanoparticles are combined with carbon nanofibers, the real and imaginary values of magnetic conductive showed obvious trend changes in the test range, these values of Co/PCNF display drastic variations with frequency increasing as demonstrated in Fig. 7d-e. In Fig. 7d, CoNPs have higher values of μ′ than Co/PCNF when the frequency is in the range of 2.0–9.5 GHz and 15.8–18.0 GHz. However, the μ′ values of Co/CPNFs are greater than that of CoNPs in the 9.5–15.8 GHz frequency range, and shows a peak value of 1.37 in the vicinity of 13.4 GHz. As shown in Fig. 7e, the value of μ″ decreases sharply as the frequency increases, and is negative in the range of 10.4–12.9 GHz. The value of μ″ is a negative number, which means that the material has generated magnetic energy and radiated outward due to charge hopping and migrating. This phenomenon may be caused by the Fabry–Perot resonance, which also appears in such as Fe_3_O_4_/SnO_2_ nanorods^30^, MWCNT/wax^31^, and hollow cobalt nanochain composites^32^. When the frequency is increased to 12.5 GHz, the μ″ value starts to rise, and clearly see the resonant frequency of the μ″ appears at 14.3 GHz.

Dielectric loss (tanδ_ε_) are the best evaluation criteria for the dielectric capacities of material. Therefore, it is necessary to lucubrate to it. As depicted in Fig. 7c, we can see that the tendency of tanδ_ε_ correspondingly in agreement with *ε*″ over a wide range of frequency, which has a sharp decline after 12.0 GHz and an upward tendency after 14.9 GHz. This is because of the value of tanδ_ε_ proportional to the value of ε″ through an equation of tanδ_ε_ = ε″/ε′. With the increase of frequency, the tanδ_ε_. The value of Co/PCNF is larger than that of CoNPs, showing Co/PCNF have a very strong dielectric loss in a certain frequency. Similarly, for Co/PCNF, the negative value of tan*δ*_ε_ in 13.7–16.9 GHz also proves that there is no dielectric loss in this frequency range. Many researchers highlight the importance of magnetic loss (tanδ_μ_) in the analysis of material magnetic loss, in an attempt to achieve the most suitable matching impedance^33^. In Fig. 7f, the tanδ_μ_ value of Co/PCNF declines from 1.16 to 0.05 in the range of 2.0–12.0 GHz, and then increases to 0.28 at about 14.7 GHz, that’s the same trend as *μ*″. However, the tanδ_μ_ value of CoNPs is almost constant around 0.05 in the entire measurement frequency range.

**Figure 8:**
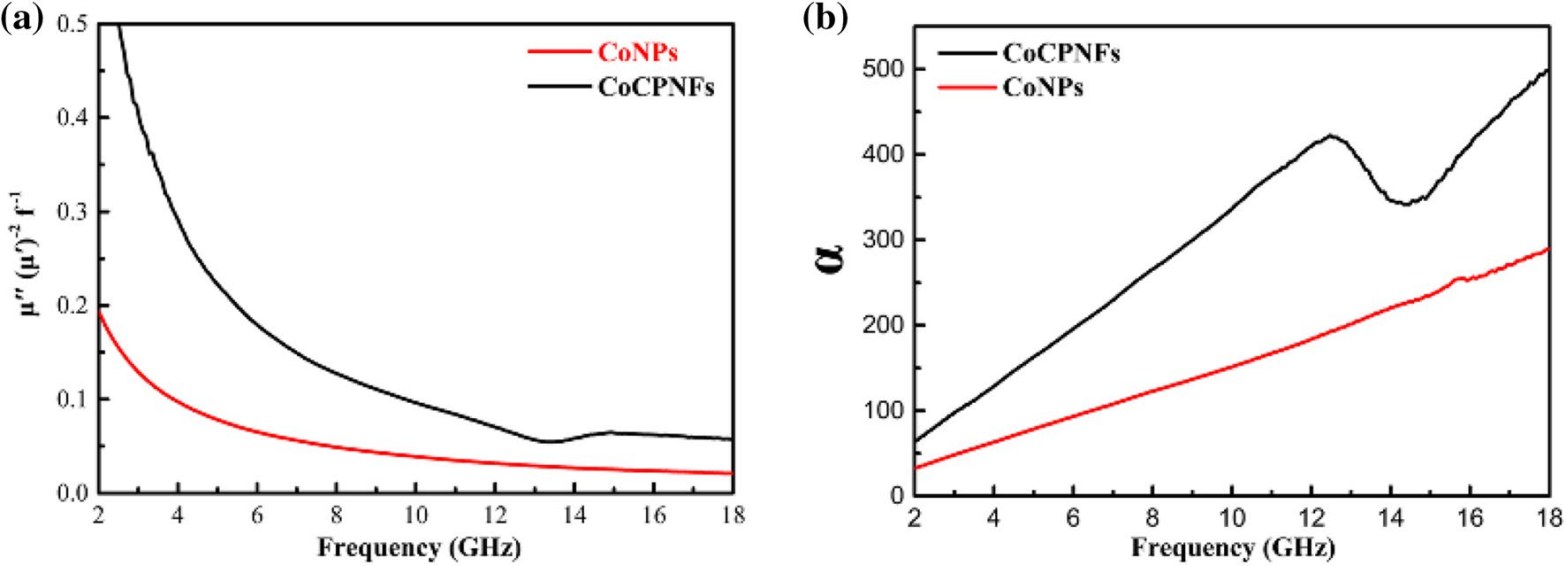
(**a**) The values of μ″(μ′)^−2^f^−1^and (**b**) the values of attenuation constant (α) versus the frequency for Co/PCNF and CoNPs.

It is well concluded that the permeability of absorber is associated with hysteresis loss, eddy current effect, domain-wall resonance, natural ferromagnetic resonance, and exchange resonance^34–37^. In the case of the weak applied microwave field, the hysteresis loss can be neglected. The domain-wall resonance loss only happens in multidomain magnetic materials instead of single domain magnetic materials. In this work, CoNPs used are multidomain magnetic materials whose particle size (Ds = 40.0 nm) is far greater than critical size of the single magnetic domain (14 nm)^38^. However, the domain-wall resonance loss can also be excluded because the domain-wall resonance loss usually exists in a much lower frequency (MHz) range^39^. In this study, due to the unique structural features and the small size of the sample, the increase in magnetic loss caused the sample to generate eddy current loss. In the test frequency range, the contribution of eddy current effect to magnetic loss can be analyzed by the dependence of c_0_ = μ″(μ′)^−2^/f with frequency^40^. If the value of c_0_ keep a constant with increasing frequency, the eddy current loss originated from eddy current effect will be the only reason for the magnetic loss^41^. It is clear that the μ″(μ′)^−2^/f values of CoNPs and Co/PCNF decrease as frequency increases is a sketch in Fig. 8a, which that means that the eddy current loss is effectively suppressed due to the smaller size of Co particles. Therefore, the eddy current effect is not the only source of magnetic loss, and also two magnetic loss mechanisms of natural resonance, exchange resonance, which work together in low frequency range^42,43^. The natural resonance can be described by the natural-resonance equation as follows:

2 $$ {2}\uppi {\text{f}}_{{\text{r}}} {\text{ = rH}}_{\upalpha } $$

3 $$ {\text{H}}_{\upalpha } { = 4}\left| {{\text{K1}}} \right|/{3}\upmu _{{0}} {\text{M}}_{{\text{s}}} $$

where r was the gyromagnetic ratio, Ha was the anisotropy energy and $\left| {{\text{K1}}} \right|$ was the anisotropy coefficient. The natural ferromagnetic resonance may be attributed to the small size effect and the introduction of non-magnetic materials. In Co/PCNF, due to the surface anisotropic field generated by the small size effect of the material itself, this effect is significantly enhanced by the nano-scale CoNPs prepared by HEIBE. Not only that, when non-magnetic materials are introduced, the MS of the sample is lower than the Ms of CoNPs, which makes the anisotropy energy of the samples higher than anisotropy energy of CoNPs. The higher anisotropy energy of the sample is beneficial to improve the microwave absorption performance. As illustrated in Fig. 7e,f, the μ″ and tanδ_μ_ curves of Co/PCNF present strong peaks near 14.5 GHz, and the absorption peak caused by natural resonance and exchange resonance appear in the high-frequency range^44,45,54^.

To the best of our knowledge, the attenuation constant (α) is also an important factor affecting reflection loss, which is widely utilized to demonstrate the intrinsic loss ability of MAMs. Depending on transmission line theory and electromagnetic wave propagation constant, attenuation constant (α) can be defined as^46^:

4 $$ \upalpha  = \frac{{\sqrt 2\uppi {\text{f}}}}{{\text{c}}} \times \sqrt {\left( {\upmu ^{^{\prime\prime}}\upvarepsilon ^{^{\prime\prime}} -\upmu ^{\prime }\upvarepsilon ^{\prime } } \right) + \sqrt {\left( {\upmu ^{^{\prime\prime}}\upvarepsilon ^{^{\prime\prime}} -\upmu ^{\prime }\upvarepsilon ^{\prime } } \right)^{2} + \left( {\upmu ^{\prime }\upvarepsilon ^{^{\prime\prime}} -\upmu ^{^{\prime\prime}}\upvarepsilon ^{\prime } } \right)^{2} } } $$

As frequency aggrandize, both the attenuation constant (α) of CoNPs and Co/PCNF increase (Fig. 8b). It is obvious that after adding carbon nanofiber to combined with CoNPs, the attenuation constant (α) of Co/PCNF is far greater than CoNPs. Hence, it is concluded that the dielectric attenuation plays a predominant role in the final microwave absorption behavior, which mainly resulted from conductive loss, dipole polarization, and interfacial polarization^47^. This result indicates that Co/PCNF have superior attenuation ability for the incident electromagnetic waves.

It is well known that when electromagnetic waves are incident on the surface of the absorber, part of the electromagnetic wave energy will inevitably be reflected by the surface of the material, and the other part will enter the inside of the absorber and be attenuated by the material. The energy of the electromagnetic wave reflected by the surface is determined by the reflection factor (Γ), which can be expressed by a formula composed of the input characteristic impedance (Z_in_) and the characteristic impedance of free space (Z_0_). According to the line transmission theory, using MATLAB to calculate the reflection loss (RL) value of the composite electromagnetic parameters and the thickness of the absorber, the calculation formula is as follows^47–50^:

5 $$ \Gamma = {\text{(Z}}_{{{\text{in}}}} - {\text{Z}}_{{0}} {\text{)/(Z}}_{{{\text{in}}}} + {\text{Z}}_{{0}} {)} $$

6 $$ {\text{RL}}\left( {{\text{dB}}} \right) = 20{\text{log}}\left| {\frac{{{\text{Z}}_{{{\text{in}}}} - {\text{Z}}_{0} }}{{{\text{Z}}_{{{\text{in}}}} + {\text{Z}}_{{0}} }}} \right| $$

7 $$  {\text{Z}}_{{{\text{in}}}}  = {\text{Z}}_{0} \sqrt {\frac{{{\upmu }_{{\text{r}}} }}{{{\upvarepsilon }_{{\text{r}}} }}} {\text{tanh}}\left[ {{\text{j}}\frac{{{\text{2}}{\uppi }{\text{ft}}}}{{\text{c}}}\sqrt {{\upmu }_{{\text{r}}} {\upvarepsilon }_{{\text{r}}} } } \right]  $$

where Z_in_ is the input impedance of the absorber, Z_0_ is the impedance of free space, μ_r_ and ε_r_ is the relative complex permeability and permittivity, respectively, f is frequency of the electromagnetic wave, c is velocity of electromagnetic waves in free space, and t is thickness of an absorber.

**Figure 9:**
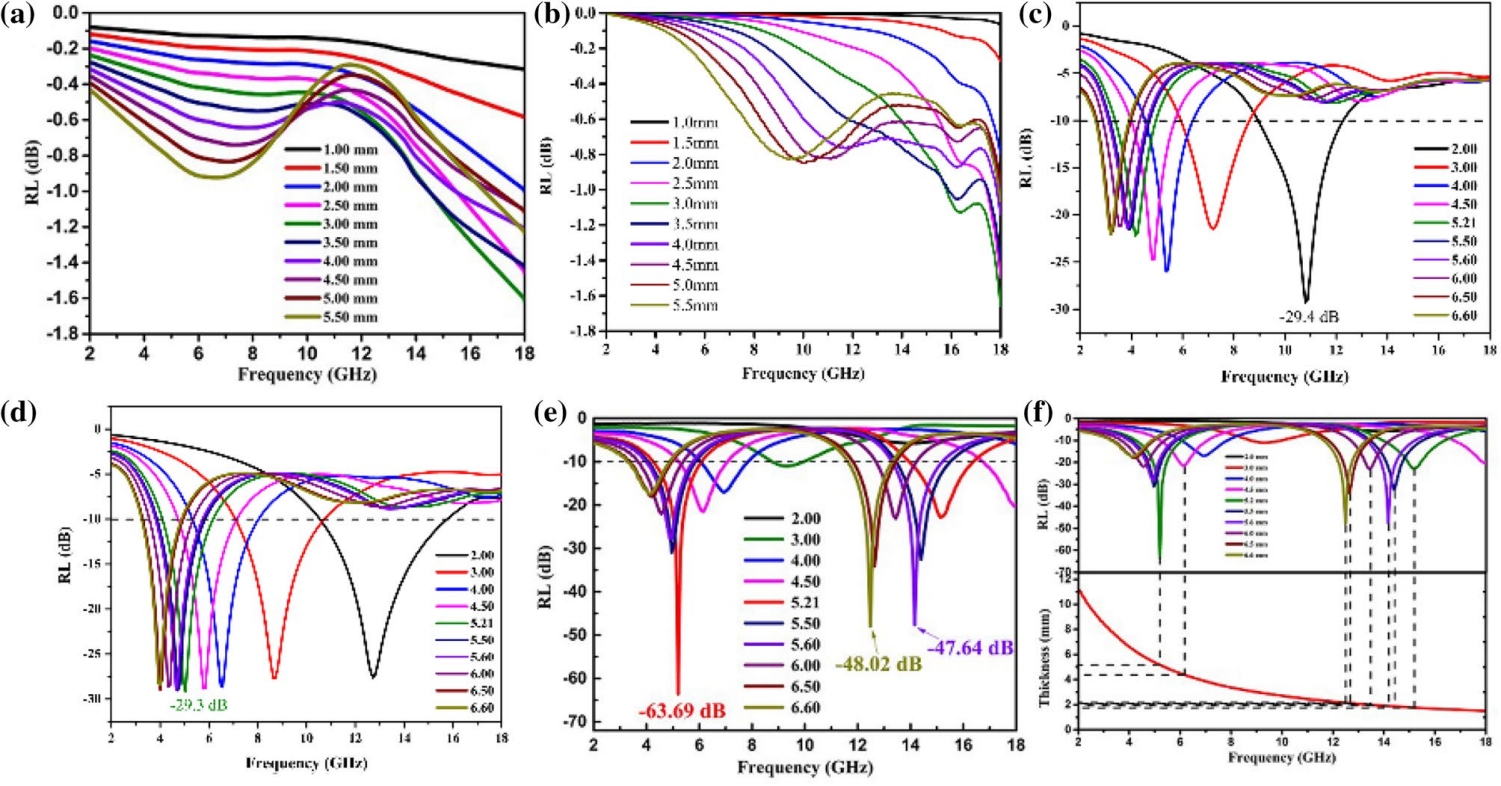
(**a**) Reflection loss (RL) curves for CoNPs, (**b**) Reflection loss (RL) curves for PCNF, (**c**) S1, (**d**) S2 and (**e**) S3 with different thickness in the frequency range of 2–18 GHz, (**f**) RL values of Co/PCNF at “matching thickness” (tm) in 2.00–18.00 GHz.

**Figure 10:**
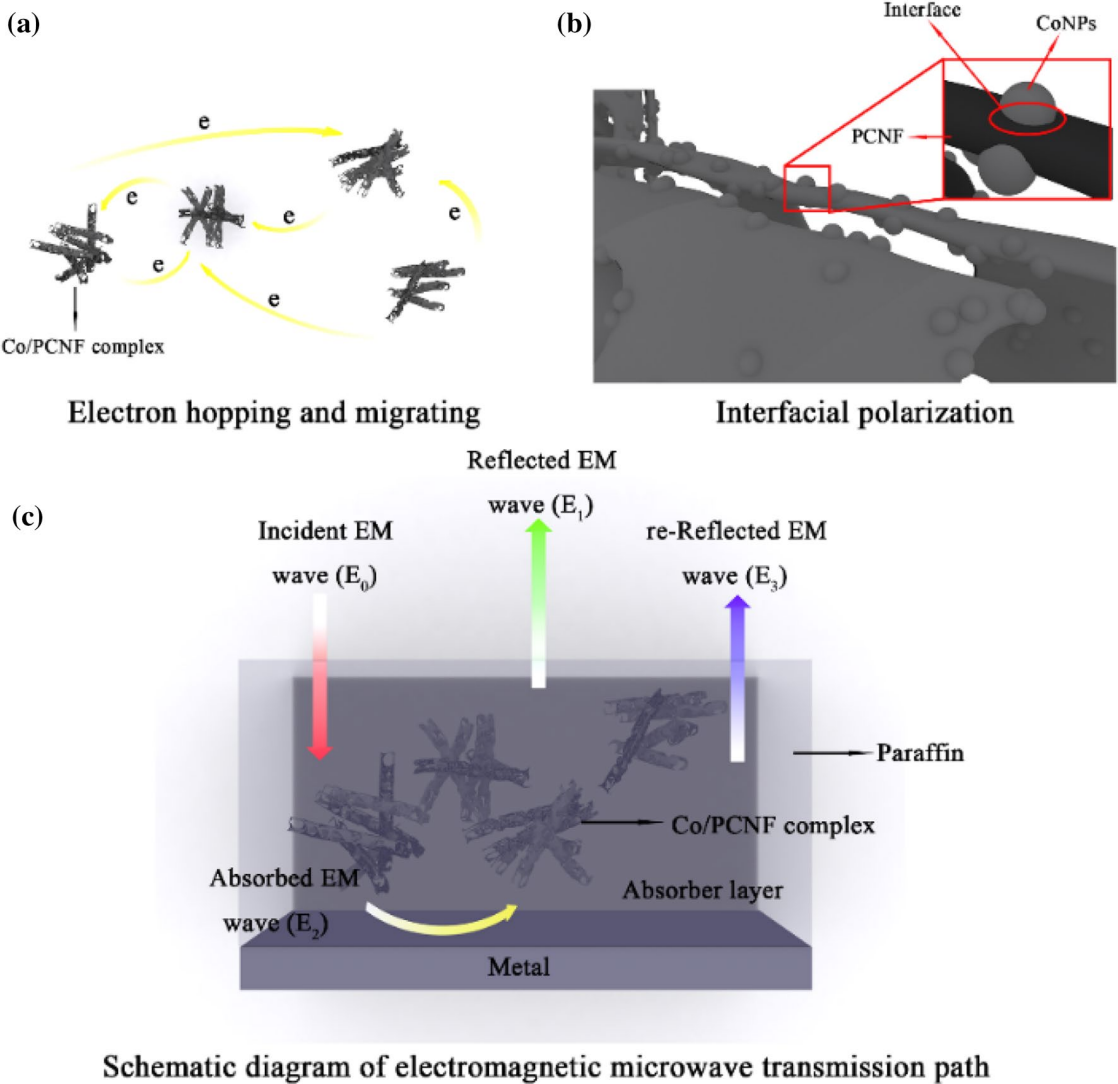
The illustration for main microwave attenuation mechanisms. (**a**) Hopping and migrating of free electrons under an electromagnetic field, (**b**) Interface polarization at the interface of CoNPs and PCNF, (**c**) EM transmission path.

The reflection loss (RL) curves of CoNPs, PCNF, and Co/PCNF at different thickness in 2.00–18.00 GHz is sketched in Fig. 9a–f. The reflection loss (RL) of a single materials CoNPs is disappointing, the maximum reflection loss (RL) value of it is only – 1.60 dB at 18.00 GHz with a thickness of 3.00 mm, as shown in Fig. 9a. For the sake of contrast, this work also gives the RL value of PCNF (Fig. 9b). Obviously, both PCNFs and CoNPs are single materials, and their RL values are not ideal, and they cannot be applied solely to the absorption of EM. Correspondingly, Co/PCNF (Fig. 9c–e) exhibits an excellent microwave absorption property: As the content of CoNPs increases, the absorbing performance of Co/PCNF shows a trend of first increasing and then decreasing. As the content of CoNPs increases, the absorbing performance of Co/PCNF shows a trend of first increasing and then decreasing. The effective absorption bandwidth of S1 in the entire test range is 9.4 GHz (2.6–8.6 GHz, 8.9–12.3 GHz), and the minimum reflection value reaches − 29.4 dB at a thickness of 2.00 mm (as shown in Fig. 9c). In contrast, the performance of S2 is relatively excellent (as shown in Fig. 9d), the effective absorption bandwidth in the range of 2–18 GHz reaches 12.5 GHz (8.9–12.3 GHz), and the reflection value reaches − 29.3 GHz when the absorbing body thickness is 5.21 mm. Among all the samples, the best performance is S3. In Fig. 9e, when the absorber thickness exceeds 3 mm, the composite materials begin to affect electromagnetic wave attenuation performance (the RL value is less than − 10 dB, more than 90% attenuation), and maximum reflection loss (RL) value can reach − 63.69 dB at 5.28 GHz with a thickness of 5.21 mm. Furthermore, as for 5.60 mm and 6.61 mm absorber, there are two absorption peaks of − 47.60 dB and − 48.30 dB appear around 12.50 GHz and 14.10 GHz, respectively. At the same time, the effective absorption bandwidth (EABW, RL ≤  − 10.0 dB) of Co/PCNF is 12.92 GHz (3.18–7.96 GHz and 11.55–18.00 GHz). Thus, the S3 absorber can effectively attenuate the EM wave in part of the C band and entire K_u_ band. It's clear that the maximum reflection loss (RL) value of Co/PCNF moves to the low frequency band with a decrease in thickness, where the show in Fig. 9f. This phenomenon can be mainly explained by the theory of quarter-wavelength, which can be expressed by the following equation^51,53^:

8 $$ {\text{t}}_{{\text{m}}} = {\text{n}}/4 = {\text{nc}}/\left( {4{\text{f}}_{{\text{m}}} \sqrt {\left|\upmu \right|\left|\upvarepsilon \right|} } \right)\quad \left( {{\text{n}} = 1,3,5 \ldots } \right) $$

where c is the velocity of light. When the thickness of the absorber satisfies the quarter-wavelength cancellation model, the phase difference between the incident electromagnetic wave and the reflected electromagnetic wave isπ, and they will cancel each other on the air-material surface, which is beneficial to the improvement of the absorption performance (as shown in Fig. 10c). This kind of variation tells us that the microwave absorption properties of Co/PCNF can be regulated by matching the absorber thickness in a frequency range of 2.00–18.00 GHz. Figure 9d shows that t_m_ calculated by MATLAB can perfectly be matching the RL curve, indicating that Co/PCNF satisfies the quarter-wavelength formula.

We further evaluate the microwave absorption performance (reflection loss, absorber thickness, and absorption bandwidth) of Co/PCNF in comparison to those of the same types of Co/C composites ever reported (Table 2).

**Table 2:** Microwave absorption performance of various typical Co-based composites.

Sample	Maximum RL			Absorber thickness (RL ≤ − 10 dB) (mm)	Absorption bandwidth (RL ≤ − 10 dB) (GHz)	Refs.
	Value (dB)	Matching thickness (mm)	Matching frequency (GHz)			
Co/CNFs	− 63.10	1.60	12.80	1.10–5.00	14.30 (3.70–18.00)	^5^
Co-CNTs	− 39.32	3.00	15.71	3.00–4.00	5.70 (11.80–17.50)	^6^
Co/C nanoparticles	− 43.40	2.30	16.80	1.00–4.00	14.00 (4.00–18.00)	^7^
Co/C nanocomposites	− 35.30	2.50	5.80	1.00–5.00	13.70 (3.70–17.40)	^8^
Co/Graphene	− 47.50	2.00	11.90	1.00–5.00	11.20 (2.80–14.00)	^9^
Co/C-800	− 32.40	2.00	9.60	1.00–5.00	13.50 (4.50–18.00)	^10^
Co/Porous carbon	− 40.00	5.00	4.20	1.50–5.00	12.80 (3.70–16.50)	^34^
Co/CPNFs	− 63.69	5.21	5.28	1.00–6.61	12.92 (3.2–018.00)	Herein

Ultimately, the dielectric loss a part of excellent microwave absorption performance of Co/PCNF comes of different resistivity of carbon materials and magnetic nanoparticles, which leads to electrons asymmetry distribution, then a large amount of space charges is accumulated in the interface, eventually leading to the occurrence of interface polarization^51,52,55^. Structurally, the stronger interface polarization existed in the carbon-porous interface and the CoNPs-porous interface by the porous is abundant in Co/PCNF. In addition, when the EM wave enters the absorber, the unique 3D cross-linked network structure can provide multiple reflections. Moreover, benefited from the relatively low graphitization degree, Co/PCNF obtained excellent matching impedance, which can reduce the reflection of the material surface to EM wave and will allow more microwave can be transmitted into the materials. Above all, Dielectric loss is mainly caused by conductance loss and interfacial polarization. Natural resonance, exchange resonance, and eddy current loss provide magnetic loss mechanism. As a result, it is that we can design the impedance match and interfacial polarization of absorber by introducing the porous nanostructure, which plays a positive role in microwave absorption. Thus, due to the above reasons, the Co/PCNF has potential to be a microwave absorbing material with superior attenuation ability for the incident electromagnetic waves.

## Conclusions

In general, we used high energy ion beam evaporation (HEIBE) to prepare the core–shell structure cobalt nanoparticles (CoNPs), and the Co/C porous nanofibers (Co/CPNFs) with low graphitization degree obtained by the electrospinning method and following sintering process with sintering temperature of 800 ℃. Experimental results show that by 3D-network structure, porous structure and magnetic nanoparticles doping, the impedance matching, dielectric loss, magnetic loss, and high attenuation constant can be optimized, which leads to Co/CPNFs have excellent microwave absorption properties. Beyond that, the porous and Co nanoparticles also can introduce a new interface, defects to enhance interfacial polarization. The Co/CPNFs exhibit the maximum reflection loss (RL) reaches − 63.69 dB at 5.28 GHz with a thickness of 5.21 mm and the absorption bandwidth (RL ≤ − 10.0 dB) is 12.92 GHz (3.18–7.96 GHz and 11.55–18.00 GHz). These results suggest that Co/C porous nanofibers are considered to be a suitable candidate for the manufacture of lightweight and high-efficiency microwave absorbing materials. Also, the facile synthesis of Co/CPNFs provide an easy and implemental method for large scale preparation of high-performance microwave absorption materials.
